# Short daytime napping reduces the risk of cognitive decline in community-dwelling older adults: a 5-year longitudinal study

**DOI:** 10.1186/s12877-021-02418-0

**Published:** 2021-08-28

**Authors:** Kaori Kitamura, Yumi Watanabe, Kazutoshi Nakamura, Chikako Takano, Naomi Hayashi, Hisami Sato, Toshiyuki Someya

**Affiliations:** 1 Division of Preventive Medicine, Niigata University Graduate School of Medical and Dental Sciences, Niigata, 951-8510 Japan; 2 Ojiya City Government, Ojiya, Niigata, 947-0028 Japan; 3 Department of Psychiatry, Niigata University Graduate School of Medical and Dental Sciences, Niigata, 951-8510 Japan

**Keywords:** Cohort studies, Napping, Sleep, Cognitive decline, Dementia, Epidemiology, Preventive medicine

## Abstract

**Background:**

Beneficial effects of napping on cognition have been suggested in cross-sectional studies. This study aimed to clarify longitudinal associations between cognitive decline and sleep characteristics, particularly daytime napping, over a 5-year period in older adults.

**Methods:**

Study participants were 389 community-dwelling individuals aged ≥65 years living in Ojiya City, Niigata, Japan. Baseline and follow-up examinations were conducted in 2011–2013 and 2016–2018, respectively. Trained nurses visited and interviewed participants to collect the following information at baseline and follow-up: demographic characteristics, disease history, lifestyle habits including bedtime, sleeping hours, and daytime nap duration, and cognitive function. The assessment of cognitive function was performed using the revised Hasegawa’s dementia scale (HDS-R), with cognitive decline defined as a change in the HDS-R of ≤ − 3 over 5 years. Odds ratios (ORs) for cognitive decline were calculated using multiple logistic regression analysis.

**Results:**

Mean age of participants was 74.6 years (SD 6.4), and the cumulative incidence of cognitive decline was 106/389 (27.3%). The adjusted OR for 1–29 min daytime napping was significantly lower compared to that for no napping (OR = 0.47, 95%CI: 0.23–0.96). Earlier bedtime was associated with cognitive decline (adjusted P for trend = 0.0480).

**Conclusion:**

Short daytime napping (< 30 min) reduces the risk of cognitive decline over 5 years for community-dwelling older people. A future study will be necessary to confirm the effect of short napping on the reduction of risk for clinically diagnosed dementia.

**Supplementary Information:**

The online version contains supplementary material available at 10.1186/s12877-021-02418-0.

## Background

Dementia places a tremendous burden on society worldwide. The total number of people with dementia in the world was estimated to be 35.6 million in 2010, and this number is projected to increase to 115.4 million in 2050 [1]. The total cost of dementia is also enormous, estimated at US$ 604 billion in 2010 [1]. Under these circumstances, the prevention of dementia and dementia-related disorders is of high priority.

The role of sleep in cognitive function and dementia has drawn attention, although evidence is still insufficient [2]. According to recent reviews and meta-analyses, sleep duration and sleep disturbance are determinants of cognitive decline and dementia [3–7]. Moreover, daytime napping is reportedly associated with cognitive function in older adults [8–11]. However, findings from previous studies have been somewhat inconsistent; some reported possible adverse effects of napping, especially long napping, on cognitive function [9, 11], whereas others reported possible beneficial effects of napping, especially short napping [8, 10]. Furthermore, except for one longitudinal study [8], only cross-sectional studies [9–11] have been conducted.

We previously conducted an epidemiologic study to investigate associations between cognitive impairment and lifestyle factors, including sleep characteristics and daytime napping, in community-dwelling older adults [12]. The present study aimed to clarify longitudinal associations between cognitive decline and sleep characteristics, in particular whether daytime napping is beneficial or harmful for cognitive function, based on 5-year follow-up data from participants of the study mentioned above.

## Methods

### Design and participants

**Fig. 1:**
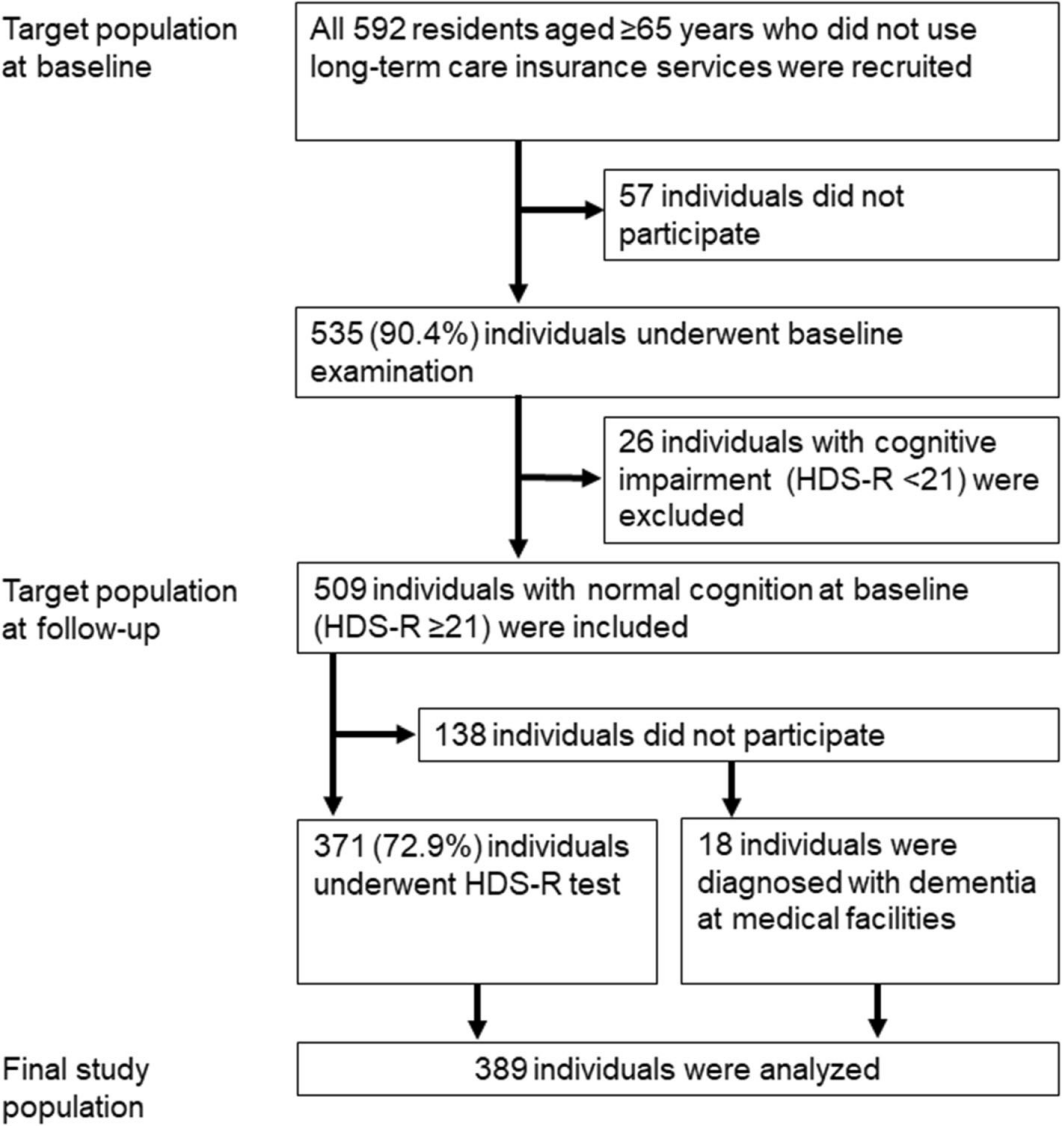
Flow chart of participant enrollment

This study was a 5-year follow-up cohort study. Participants of the baseline study were included in the study, and those with cognitive impairment diagnosed by the revised Hasegawa’s dementia scale (HDS-R) at baseline were excluded. Participants at baseline were community-dwelling older adults living in the following three areas of Ojiya City, Niigata, which were set by the city government as model areas: Heiseicho (an urban area), Matto (a rural, farming area), and central Katakai (an urban area) [12]. Among all 592 residents aged ≥65 years who were not receiving long-term care insurance services and who were invited to participate in the study, 535 (90.4%) underwent the baseline examination. A high participation rate (90.4%) was obtained due to efforts of public health nurses in charge of each area. Of these 535 residents, 509 (95.1%) who were considered cognitively normal were invited to participate in the present 5-year follow-up study, and 371 (72.9%) underwent the follow-up examination. We also included 18 individuals who had normal cognitive function at baseline and did not participate in the follow-up examination, but were diagnosed with dementia at medical facilities during the follow-up period, because these individuals met our diagnostic criteria of cognitive decline described below. The final study cohort thus comprised 389 individuals. Figure 1 shows the flow of participant enrollment. Informed consent was obtained from all participants who underwent the follow-up examination. The consent was verbal because, according to the Ethical Guidelines for Medical and Health Research Involving Human Subjects in Japan [13], investigators are not required to obtain informed consent in writing for human studies which are not invasive or do not involve interventions. The protocol of this study was approved by the Ethics Committee of Niigata University.

### Baseline examination

The baseline examination was conducted in three areas of Ojiya city in October–December 2011 for Heisei-cho residents, August–November 2012 for Matto residents, and July–September 2013 for Katakai residents. Trained nurses visited and interviewed participants to collect the following information: demographic characteristics, health status (including cognitive function) and lifestyle, family environment (living with family or alone), current occupational status (unemployed/retired or employed), and histories of hypertension, cerebrovascular disease, and diabetes. The interviewer’s guide of this study is shown in Additional Information 1. Cognitive function was assessed using the HDS-R [14]. We also collected information regarding alcohol consumption (classified into five categories: 1) non-drinker, 2) < 7 *gou* (1 *gou* is equivalent to 180 mL of Japanese *sake*), 3) 7–13 *gou*, 4) 14–20 *gou*, and 5) ≥21 *gou* per week) and smoking status (classified into three categories: 1) non-smoker, 2) past smoker, and 3) current smoker). Usual bedtime and sleeping hours were asked and recorded, and the duration of daytime napping was recorded as 1) none, 2) < 30 min, 3) 30–59 min, and 4) ≥60 min. We did not consider the number of daytime naps because this information was not obtained during the interviews. Participants were also asked about current sleep disturbances and the use of sleeping pills. Details of the baseline examination have been described previously [12].

### Five-year follow-up examination

The follow-up examination, including the HDS-R test, was conducted 5 years after, and in the same manner as, the baseline examination, i.e., in October–December 2016 for Heisei-cho residents, August–November 2017 for Matto residents, and July–August 2018 for Katakai residents.

### Assessment of cognitive function

The HDS-R, a 30-point test, was used to assess general cognitive function, with a score ≤ 20 defined as cognitive impairment at baseline. The HDS-R was developed to screen for dementia (sensitivity: 0.90, specificity: 0.82) with a cutoff of 20/21 [14]. The HDS-R has been used in East Asian populations [15, 16] and demonstrated to have a diagnostic accuracy similar to that of the MMSE [17]. One advantage of using the HDS-R over the MMSE is its diagnostic accuracy regardless of education level [17]. The HDS-R was administered during baseline and follow-up examinations, and change in HDS-R (ΔHDS-R = [score at follow-up] – [score at the baseline]) was calculated. We used a cutoff of ΔHDS-R ≤ -3 to detect the presence of cognitive decline, referring to the cutoff of ΔMMSE ≤-3 (30-point test) used in previous studies [18, 19], based on the fact that longitudinal scores of HDS-R and MMSE change in the same direction in community-dwelling individuals [20]. We also included 18 individuals who had normal cognitive function at baseline and did not participate in the follow-up examination, but were diagnosed with dementia at medical facilities, as having cognitive decline.

### Statistical methods

The χ^2^ test was used to test for independence of categorical data for participant characteristics. Cumulative incidence of cognitive decline was calculated and compared according to levels of potential predictor variables by odds ratios (ORs) computed using simple and multiple logistic regression analyses. Dose-dependent associations were assessed by simple and multiple logistic regression analyses and reported as P for trend values. First, unadjusted ORs for cognitive decline according to potential predictors were calculated. Second, ORs were adjusted for age and baseline HDS-R. Third, we calculated multivariable-adjusted ORs (hereafter referred to as “fully-adjusted ORs”) of bedtime, duration of sleep, and duration of nap for cognitive decline using the forced entry method to adjust for the confounding effects of all potential predictor variables. For example, the OR for duration of sleep was calculated and adjusted for age, baseline HDS-R, sex, region (dummy variables), family environment, job status, histories of hypertension, cerebrovascular diseases, and diabetes, alcohol consumption, smoking status, bedtime, duration of nap, presence of sleep disturbance, and use of sleeping pills. Duration of nap was transformed into a dummy variable (0, 1–29 min; 1, others) because the results suggest that a 1–29 min nap is associated with a decreased risk of cognitive decline. Although a cutoff of ΔHDS-R ≤ -3 was used to detect cognitive decline, we used cutoffs of ≤ − 4 and ≤ − 2 for the sensitivity analyses. Outliers exceeding 3SD were not found among continuous variables. SAS statistical software (release 9.1.3, SAS Institute Inc., Cary, NC, USA) was used for data analysis. *P* < 0.05 was considered statistically significant.

## Results

The mean age of participants was 74.6 years (SD 6.4). Table 1 shows the baseline characteristics of participants by sex. Significant sex-based differences were observed in family environment, job status, and alcohol consumption. Specifically, significantly higher proportions of women lived alone, were unemployed, had lower alcohol consumption, and had a later bedtime than men. Moreover, women took significantly shorter naps than men, and had a greater proportion of those who do not nap at all.

**Table 1:** Participant characteristics at baseline

Characteristics	Men (*n* = 159)	Women (*n* = 230)	*P* value
Age (years)			
65–69	39 (24.5%)	58 (25.2%)	0.9955
70–79	83 (52.2%)	117 (50.9%)	
80–89	35 (22.0%)	52 (22.6%)	
90–99	2 (1.3%)	3 (1.3%)	
Baseline HDS-R score			
≥ 26	126 (79.3%)	195 (84.8%)	0.1575
21–25	33 (20.8%)	35 (15.2%)	
Area of residence			
Heisei-cho	40 (25.2%)	63 (27.4%)	0.8174
Matto	67 (42.1%)	98 (42.6%)	
Katakai	52 (32.7%)	69 (30.0%)	
Family environment			
Living with family	153 (96.2%)	206 (89.6%)	0.0155
Living alone	6 (3.8%)	24 (10.4%)	
Job status			
Employed	84 (52.8%)	52 (22.6%)	< 0.0001
Unemployed/retired	75 (47.2%)	178 (77.4%)	
History of hypertension			
Absent	82 (51.6%)	116 (50.4%)	0.8254
Present	77 (48.4%)	114 (49.6%)	
History of cerebrovascular disease			
Absent	150 (94.3%)	224 (97.4%)	0.1244
Present	9 (5.7%)	6 (2.6%)	
History of diabetes			
Absent	146 (91.8%)	210 (91.3%)	0.8565
Present	13 (8.2%)	20 (8.7%)	
Alcohol consumption^a^(*gou*/wk)			
Non-drinker	41 (25.8%)	167 (72.6%)	< 0.0001
< 7	21 (13.2%)	37 (16.1%)	
7–13	36 (22.6%)	22 (9.6%)	
14–20	41 (25.8%)	3 (1.3%)	
≥ 21	20 (12.6%)	1 (0.4%)	
Smoking			
Non-smoker	53 (33.3%)	224 (97.4%)	< 0.0001
Past smoker	60 (37.7%)	2 (0.9%)	
Current smoker	46 (28.9%)	4 (1.7%)	
Bedtime			
-8:59 p.m.	27 (17.0%)	18 (7.8%)	< 0.0001
9:00–9:59 p.m.	62 (39.0%)	54 (23.5%)	
10:00–10:59 p.m.	39 (24.5%)	79 (34.3%)	
11:00 p.m.-	31 (19.5%)	79 (34.3%)	
Duration of nighttime sleep (hr)			
< 6	23 (14.5%)	48 (20.9%)	0.1437
6–6.9	38 (23.9%)	65 (28.3%)	
7–7.9	52 (32.7%)	73 (31.7%)	
8–8.9	35 (22.0%)	32 (13.9%)	
≥ 9	11 (6.9%)	12 (5.2%)	
Duration of daytime nap (min)			
0	52 (32.7%)	101 (43.9%)	0.0098
1–29	35 (22.0%)	63 (27.4%)	
30–59	39 (24.5%)	35 (15.2%)	
≥ 60	33 (20.8%)	31 (13.5%)	

^a^Equivalent to Japanese *sake* (1 *gou* of *sake* is equivalent to 180 mg *sake* or 27 g ethanol)

The overall cumulative incidence of cognitive decline was 106/389 (27.3%). Table 2 shows the cumulative incidence, unadjusted ORs, and age- and baseline-HDS-R-adjusted ORs for cognitive decline according to levels of predictor variables. Age was robustly associated with unadjusted ORs for cognitive decline. The age- and baseline-HDS-R-adjusted OR was significantly lower for “1–29 (min)” daytime napping relative to no napping (reference).

**Table 2:** Cumulative incidence and odds ratios (ORs) for cognitive decline^a^ according to levels of predictor variables

Predictors	Cumulative incidence	Unadjusted OR (95% CI)	Adjusted OR^b^ (95% CI)	Fully-adjusted^c^ OR (95% CI)
Age (years)		P for trend< 0.0001		
65–69	12/97 (12.4%)	1 (Ref)		
70–79	46/200 (23.0%)	2.12 (1.06–4.21)		
80–89	45/87 (51.7%)	7.59 (3.63–15.85)		
90–99	3/5 (60.0%)	10.62 (1.61–70.22)		
Sex				
Men	46/159 (28.9%)	1 (Ref)	1 (Ref)	
Women	60/230 (26.1%)	0.87 (0.55–1.36)	0.80 (0.49–1.29)	
Area of residence				
Heisei-cho	30/103 (29.1%)	1 (Ref)	1 (Ref)	
Matto	37/165 (22.4%)	0.70 (0.40–1.23)	0.70 (0.39–1.27)	
Katakai	39/121 (32.2%)	1.16 (0.65–2.05)	1.14 (0.62–2.09)	
Family environment				
Living with family	94/359 (26.2%)	1 (Ref)	1 (Ref)	
Living alone	12/30 (40.0%)	1.88 (0.87–4.05)	1.81 (0.81–4.03)	
Job status				
Employed	23/136 (16.9%)	1 (Ref)	1 (Ref)	
Unemployed/retired	83/253 (32.8%)	2.40 (1.43–4.03)	1.78 (1.03–3.07)	
History of hypertension				
Absent	44/198 (22.2%)	1 (Ref)	1 (Ref)	
Present	62/191 (32.5%)	1.68 (1.07–2.64)	1.23 (0.76–1.99)	
History of cerebrovascular disease				
Absent	101/374 (27.0%)	1 (Ref)	1 (Ref)	
Present	5/15 (33.3%)	1.35 (0.45–4.05)	1.23 (0.40–3.80)	
History of diabetes				
Absent	99/356 (27.8%)	1 (Ref)	1 (Ref)	
Present	7/33 (21.2%)	0.70 (0.29–1.66)	0.72 (0.29–1.77)	
Alcohol consumption^d^(*gou*/wk)		P for trend = 0.3590	P for trend = 0.7898	
Non-drinker	64/208 (30.8%)	1 (Ref)	1 (Ref)	
< 7	13/58 (22.4%)	0.65 (0.33–1.29)	0.71 (0.34–1.45)	
7–13	11/58 (19.0%)	0.53 (0.26–1.08)	0.70 (0.33–1.48)	
14–20	11/44 (25.0%)	0.75 (0.36–1.58)	0.95 (0.43–2.09)	
≥ 21	7/21 (33.3%)	1.13 (0.43–2.92)	1.88 (0.68–5.18)	
Smoking		P for trend = 0.2706	P for trend = 0.0563	
Non-smoker	72/277 (26.0%)	1 (Ref)	1 (Ref)	
Past smoker	17/62 (27.4%)	1.08 (0.58–2.00)	1.31 (0.67–2.56)	
Current smoker	17/50 (34.0%)	1.47 (0.77–2.79)	1.94 (0.97–3.87)	
Bedtime		P for trend = 0.0698	P for trend = 0.1472	P for trend = 0.0480
-8:59 p.m.	13/45 (28.9%)	1 (Ref)	1 (Ref)	1 (Ref)
9:00–9:59 p.m.	38/116 (32.8%)	1.20 (0.57–2.54)	1.28 (0.58–2.85)	1.29 (0.55–3.04)
10:00–10:59 p.m.	33/118 (28.0%)	0.96 (0.45–2.04)	0.93 (0.42–2.09)	0.80 (0.32–2.00)
11:00 p.m.-	22/110 (20.0%)	0.62 (0.28–1.36)	0.71 (0.30–1.65)	0.50 (0.18–1.39)
Duration of nighttime sleep (hr)		P for trend = 0.0284	P for trend = 0.2737	P for trend = 0.7540
< 6	17/71 (23.9%)	1.00 (0.50–1.97)	1.16 (0.57–2.36)	1.33 (0.61–2.88)
6–6.9	24/103 (23.3%)	0.96 (0.52–1.78)	1.03 (0.54–1.94)	1.13 (0.57–2.24)
7–7.9	30/125 (24.0%)	1 (Ref)	1 (Ref)	1 (Ref)
8–8.9	26/67 (38.8%)	2.01 (1.06–3.81)	1.67 (0.84–3.32)	1.55 (0.74–3.26)
≥ 9	9/23 (39.1%)	2.04 (0.80–5.17)	1.73 (0.63–4.76)	1.47 (0.50–4.35)
Duration of daytime nap (min)		P for trend = 0.1085	P for trend = 0.8387	P for trend = 0.9923
None	44/153 (28.8%)	1 (Ref)	1 (Ref)	1 (Ref)
1–29	15/98 (15.3%)	0.45 (0.23–0.86)	0.46 (0.23–0.90)	0.47 (0.23–0.96)
30–59	21/74 (28.4%)	0.98 (0.53–1.82)	0.83 (0.43–1.59)	0.79 (0.40–1.58)
≥ 60	26/64 (40.6%)	1.70 (0.92–3.12)	1.11 (0.57–2.15)	1.05 (0.51–2.13)
Sleep disturbance				
Absent	91/338 (26.9%)	1 (Ref)	1 (Ref)	
Present	14/50 (28.0%)	1.06 (0.54–2.05)	1.20 (0.60–2.41)	
Use of sleeping pills				
No	81/308 (26.3%)	1 (Ref)	1 (Ref)	
Yes	25/81 (30.9%)	1.25 (0.73–2.14)	1.13 (0.64–2.00)	

^a^ΔHDS-R ≤ 3

^b^Adjusted for age and baseline HDS-R score

^c^Adjusted for age, baseline HDS-R score, sex, region (dummy variables), family environment, job status, histories of hypertension, cerebrovascular diseases, and diabetes, alcohol consumption, smoking status, bedtime, duration of sleep, duration of nap (0, 1–29 min; 1, others), presence of sleep disturbance, and use of sleeping pills

^d^Equivalent to Japanese *sake* (1 *gou* of *sake* is equivalent to 180 ml *sake* or 27 g ethanol)

**Fig. 2:**
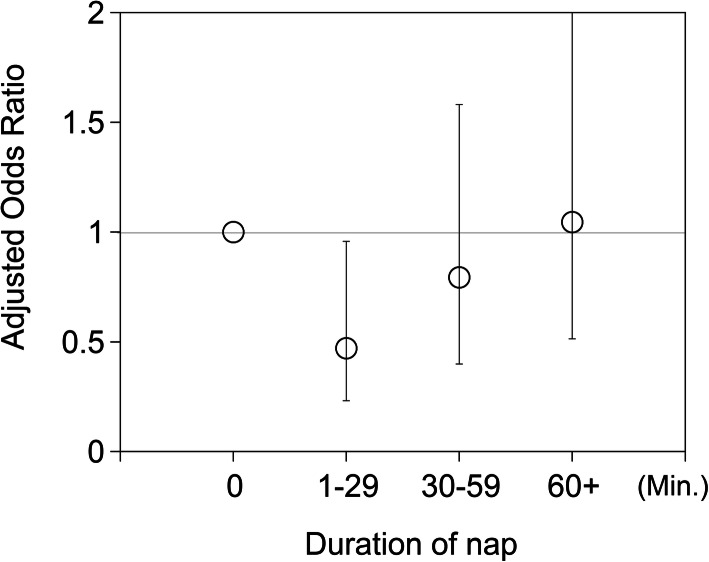
Odds ratios (ORs) for cognitive decline (ΔHDS-R ≤ -3) over 5 years according to daytime nap duration. Participants taking 1–29 min naps had a significantly lower risk of cognitive decline

The fully-adjusted OR was significantly lower for “1–29 (min)” daytime napping relative to no napping (OR = 0.47, 95%CI: 0.23–0.96) (Table 2, Fig. 2). Earlier bedtime was dose-dependently associated with cognitive decline (fully-adjusted P for trend = 0.0480) (Table 2). However, the duration of nighttime sleep was not associated with cognitive decline, sleep disturbance, or the use of sleeping pills. Napping duration and nighttime sleep duration were not significantly correlated with each other (Spearman’s correlation coefficient r = 0.082, *P* = 0.1036).

Sensitivity analyses were conducted to confirm the significant findings above. When a cutoff of ΔHDS-R ≤ -4 was used to detect cognitive decline, the fully-adjusted OR for cognitive decline was significantly lower for “1–29 (min)” daytime napping relative to no napping (fully-adjusted OR = 0.39, 95%CI: 0.16–0.91; Additional Figure 1), and the fully-adjusted P for trend of bedtime was 0.1612. When a cutoff of ΔHDS-R ≤ -2 was used, the fully-adjusted OR was marginally lower for “1–29 (min)” daytime napping relative to no napping (fully-adjusted OR = 0.58, 95%CI: 0.32–1.05; Additional Figure 2), and earlier bedtime was associated with cognitive decline (fully-adjusted P for trend = 0.0025), with a fully-adjusted OR of 0.28 (95%CI: 0.11–0.71) for “11:00 p.m.-” relative to “-8:59 p.m.” (reference).

## Discussion

This study is the first to report a significant decrease in cognitive decline in older adults who habitually take short daytime naps (< 30 min). Cross-sectional studies previously showed associations between daytime napping and cognitive impairment. Cross et al. [9] reported that longer napping is significantly correlated with increased levels of cognitive deficits in 133 adults aged > 50 years. Similarly, Owusu et al. [11] showed that unintentional, longer naps were correlated with poorer performance on cognitive tests in 2549 community-dwelling adults aged ≥65 years. While these studies suggested the unfavorable effect of longer napping, shorter napping reportedly had a favorable effect on cognitive performance [21]. In a cross-sectional study in clinical settings, Asada et al. [21] found that napping for up to 60 min, but not more than 60 min, was protective against the development of Alzheimer’s disease. Lin et al. [10] conducted a large-scale cross-sectional study in 10,740 Chinese older adults (≥60 years) and found that short nappers (< 30 min) had a significantly lower prevalence of cognitive impairment as assessed by the MMSE compared to no nappers and long nappers (≥30 min). Our findings are consistent with this report.

To date, only one longitudinal study of up to 10 years has been conducted [8], which reported that the risk of MMSE-assessed cognitive impairment was significantly lower in those who habitually take naps, regardless of duration, in a UK cohort (median age, 75 years). The discrepancy between their findings and ours may be due to the different definitions of cognitive impairment. The present study used a decrease in HDS-R score of ≤ − 3 points to define cognitive impairment, whereas Keage et al. [8] used newly diagnosed cognitive impairment according to MMSE scores. It is also possible that factors such as the difference in ethnicity might have played a role.

A number of studies have reported on the physiologically beneficial effects of napping on cognitive performance in adults [22, 23]. One study even suggested that napping leads to improved cognitive performance in older adults [24]. However, the specific effects of short naps are not fully understood. Short naps (< 30 min) reportedly improved cognitive performance and alertness and were associated with less sleep inertia [22, 24]. Moreover, an epidemiologic study found that short naps, but not long naps, had a protective effect against cardiovascular risk [23], suggesting that short naps have stress-releasing effects. These findings suggest that short naps might also be beneficial for cognitive function, since cognitive decline and dementia are considered stress-related conditions/diseases [25, 26].

The underlying mechanism by which short napping confers a beneficial effect is unclear. However, there is growing evidence that brain β-amyloid is cleared during sleep through the glymphatic system [27], and that low sleep quality is associated with brain β-amyloid burden in older adults [27]. Thus, short napping may be beneficial against brain β-amyloid by improving sleep quality. Short napping may also have a direct, favorable effect on β-amyloid clearance, although this will need to be investigated in future studies.

Recent meta-analyses have found an association between longer sleep duration and increased risk of cognitive decline [3, 4]. However, we did not find a significant association between the duration of nighttime sleep and the risk of cognitive decline, although longer sleep groups (“8–8.9-h” and “≥9-h” groups) tended to have a higher risk of cognitive decline (OR = 1.55, 95%CI: 0.74–3.26, *N* = 67, and OR = 1.47, 95%CI: 0.50–4.35, *N* = 23; respectively). The non-significant ORs may partly be due to insufficient sample sizes of the longer sleep groups. Nonetheless, our findings are consistent with the findings of the meta-analyses mentioned above [3, 4].

In the present study, an earlier bedtime was associated with a higher risk of cognitive decline (fully-adjusted P for trend = 0.0480). While evidence is scarce, a large cohort study [28] found no association between bedtime and the risk of dementia, although that study only classified bedtime as earlier or later than 11 PM. Older people living with dementia reportedly go to bed early [29]. Thus, the effect of bedtime on cognition warrants further examination.

Some baseline characteristics differed by sex, including the duration of daytime nap, in our study population. Moreover, a significantly greater proportion of women took no daytime nap compared to men, suggesting that this, as well as other female-specific characteristics (e.g., family environment, job status, alcohol consumption), may account for the higher risk of dementia in women relative to men.

The present study has several strengths. First, we used a cohort design, which is preferable for detecting causal associations. Second, this study had a high participation rate at baseline (90.4%) and an acceptable follow-up rate (72.9%). Finally, information regarding participant lifestyle was obtained and confirmed through interviews during home visits by experienced nurses.

This study also has some limitations. First, we obtained sleep-related information through interviews by trained nurses, but the information was based on self-report. Therefore, there is a possibility of misclassification bias, which might have led to an underestimation of associations between predictors and outcomes. Second, we did not collect information on naptime, although the timing of naps is an important factor related to cognitive function [24]. We did not systematically obtain information on the number of naps either. These two aspects, as well as nap duration, could influence sleep quality at night, which in turn could affect cognitive function. Finally, we did not evaluate conditions which could affect sleep, such as depression and sleep apnea.

## Conclusion

Short daytime napping (< 30 min) reduces the risk of cognitive decline over 5 years for community-dwelling older adults. Further studies will be needed to determine if short naps decrease the risk of clinically-diagnosed dementia.

## Supplementary Information

**Additional file 1: Figure 1.** Odds ratios (ORs) for cognitive decline (ΔHDS-R ≤ -4) over 5 years according to daytime nap duration.

**Additional file 2: Figure 2.** Odds ratios (ORs) for cognitive decline (ΔHDS-R ≤ -2) over 5 years according to daytime nap duration.

**Additional file 3.** The interviewer’s guide.

## Data Availability

Data are available to researchers who meet the criteria for access to confidential data. We cannot provide individual data because informed consent to provide data to anyone outside the research group was not obtained from participants. Please contact the corresponding author (principal investigator: Dr. K Nakamura) regarding any requests for access to confidential data.

## Abbreviations

HDS-R: Hasegawa’s dementia scale
ORs: Odds ratios
SD: Standard deviation
CI: Confidence interval
