# Supplementary figures and images for: Short daytime napping reduces the risk of cognitive decline in community-dwelling older adults: a 5-year longitudinal study
Source: BMC Geriatr. 2021 Aug 28;21:474. doi: 10.1186/s12877-021-02418-0 (PMC8401113; doi:10.1186/s12877-021-02418-0)

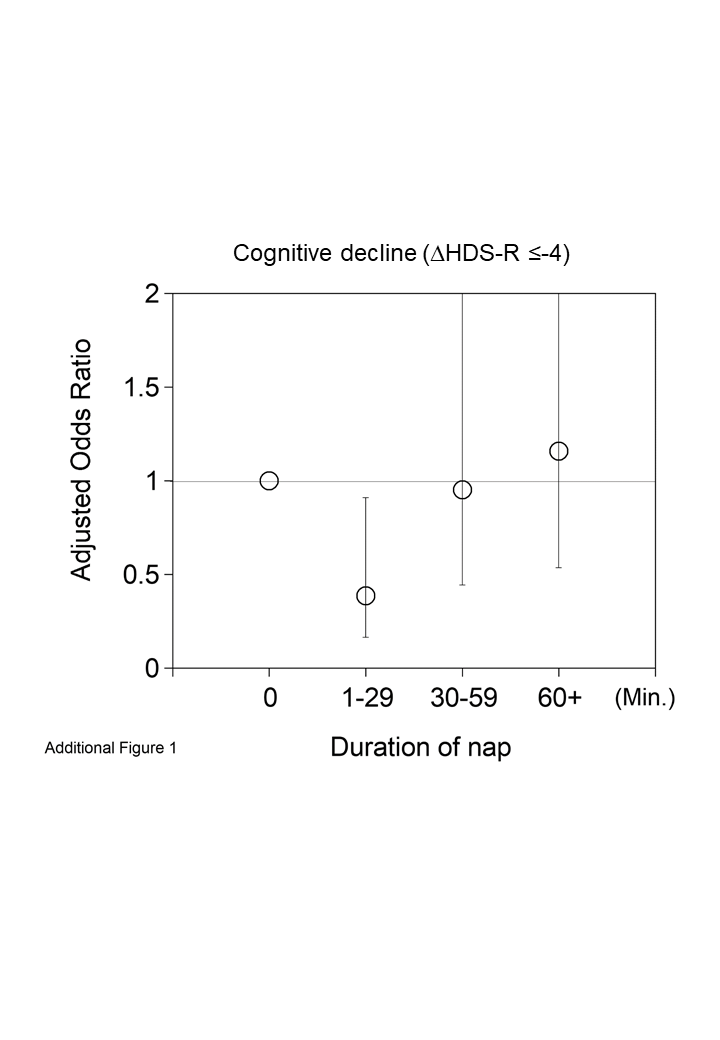

Supplement: Supplementary file 1 — Additional file 1: Figure 1. Odds ratios (ORs) for cognitive decline (ΔHDS-R ≤ -4) over 5 years according to daytime nap duration. [file 12877_2021_2418_MOESM1_ESM.tif]

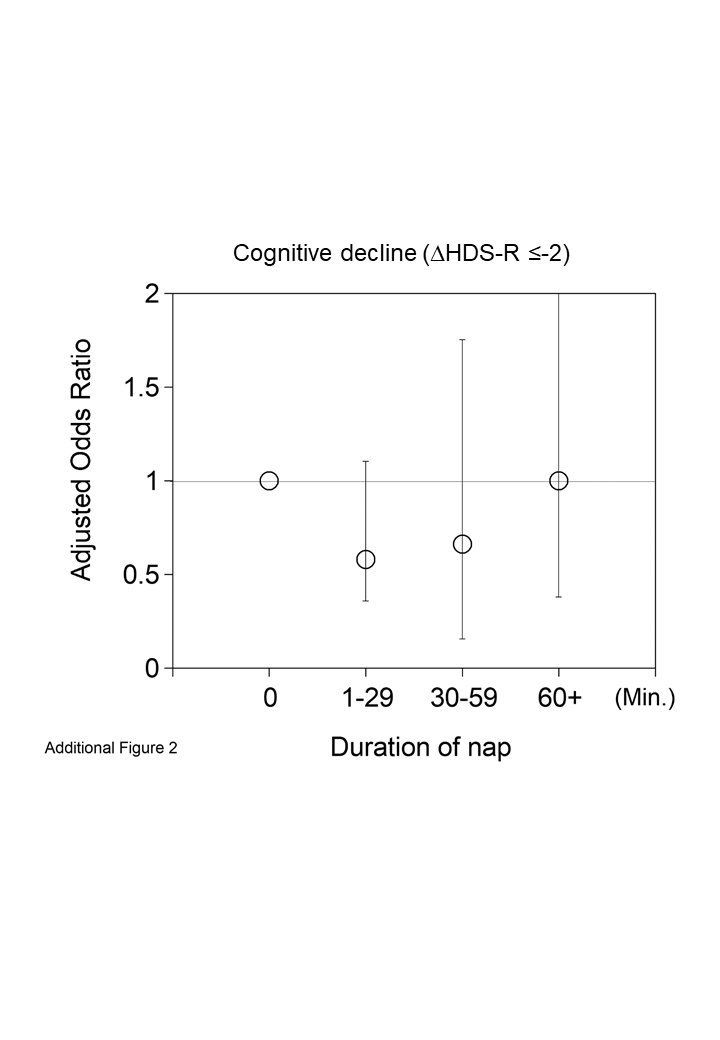

Supplement: Supplementary file 2 — Additional file 2: Figure 2. Odds ratios (ORs) for cognitive decline (ΔHDS-R ≤ -2) over 5 years according to daytime nap duration. [file 12877_2021_2418_MOESM2_ESM.tif]
