# Supplementary material for: Short daytime napping reduces the risk of cognitive decline in community-dwelling older adults: a 5-year longitudinal study
Source: BMC Geriatr. 2021 Aug 28;21:474. doi: 10.1186/s12877-021-02418-0 (PMC8401113; doi:10.1186/s12877-021-02418-0)
Supplement: Supplementary file 3 — Additional file 3. The interviewer’s guide. [file 12877_2021_2418_MOESM3_ESM.pdf]

Additional Information 1

The interviewer’s guide

*Family environment

Do you live alone or with family? Choices: (1) alone (2) with family

*Job

Do you currently have a job? Choices: (1) yes (2) no

*Disease history (based on diagnosis or prescription of medication by a doctor)

Do you have hypertension? Choices: (1) yes (2) no

Do you have a history of stroke? Choices: (1) yes (2) no

Do you have diabetes? Choices: (1) yes (2) no

*Alcohol habits

Do you drink alcohol? Choices: (1) never (2) no (ex-drinker) (3) yes

If yes, how often do you drink? Choices: (1) <1 (2) 1, 2 or 3 (3) 4, 4 or 5, 5, 6 or 7 (days/week)

If yes, how much do you drink a day?

Choices: (1) <1 (2) 1-1.9 (3) 2-2.9 (4) 3-3.9 (5) 4-4.9 (6) 5-5.9 (7) 6-6.9 (8) 7-7.9 (9) 8-8.9 (10) 9-9.9 (11) 10 or more (*gou*/days; 1 *gou* is equivalent to 180 mL of Japanese *sake*)

*Smoking habits

Do you smoke? Choices: (1) never (2) no (ex-smoker) (3) yes

*Sleep habits

What time do you go to sleep? Typical time: _____:_____ (PM or AM)

How long do you sleep? (typical sleep duration)

Choices: (1) <3 (2) 3-3.9 (3) 4-4.9 (4) 5-5.9 (5)6-6.9 (6) 7-7.9 (7) 8-8.9 (8) 9-9.9 (9) 10 or more (hours)

How is your sleep quality? Choices: (1) good (2) bad (sleep disturbance)

Do you take any sleep pills? Choices: (1) yes (2) no

Do you take a daytime nap? If so, for how long? Choices: (1) no (2) <30 (3) 30-59 (4) ≥60 (minutes)

*Cognitive function is assessed using the revised Hasegawa’s dementia scale (HDS-R) (Imai & Hasegawa, 1994).

Appendix 1. Hasegawa's Dementia Scale - Revised (HDS-R)

1.	How old are you? *(+/- 2 yrs.)*		0	1	
2.	Year, month, date, day?<br>*1 point each.*	Year<br>Month<br>Date<br>Day	0<br>0<br>0<br>0	1<br>1<br>1<br>1	
3.	What is this place?<br>*Correct answer in 5 sec.:2 points*<br>*Correct choice between "hospital? office?"*		 0<br>0	 2<br>1	
4.	Repeating 3 words. *1 point each.* *(To use only one version per test.)*<br>*Version A:"a)cherry blossom b)cat c)tram"*<br>*Version B:"a)plum blossom b)dog c)car"*	a)<br>b)<br>c)	0<br>0<br>0	1<br>1<br>1	
5.	100-7=?*If correct, 1 point.*<br>*If not:skip to item #6.*<br>-7 again=?*If correct, 1 point.*	93<br><br>86	0<br><br>0	1<br><br>1	
6.	Repeat 6-8-2 backwards.<br>*If not:skip to item #7.*<br>Repeat 3-5-2-9 backwards.		0<br><br>0	1<br><br>1	
7.	Recall 3 words. For each words<br>*2 points for spontaneous recall.*<br>*1 points for correct recall after category cue*	a)<br>b)<br>c)	0<br>0<br>0	1<br>1<br>1	2<br>2<br>2
8.	Show five unrelated common object, then take them back and ask for recall.<br>*1 point each.*		0<br>3	1<br>4	2<br>5
9.	Name all vegetables that come to mind.<br>*No time limit. May remind once.*<br>*Terminate when there is no further answer after a 10 sec. interval. For each vegetable name after the 5th one:1 point.*<br>1.______ 2.______ 3.______ 4.______ 5.______<br>6.______ 7.______ 8.______ 9.______ 10.______		0<br>3	1<br>4	2<br>5
	Total score				/30
